# The Demographic Stretch of the Arc of Life: Social and Cultural Changes That Follow the Demographic Transition

**DOI:** 10.9745/GHSP-D-14-00175

**Published:** 2015-09-10

**Authors:** Ariel Pablos-Mendez, Scott R Radloff, Kamiar Khajavi, Sally Ann Dunst

**Affiliations:** ^a^​ United States Agency for International Development, Washington, DC, USA; ^b^​ Johns Hopkins Bloomberg School of Public Health, Baltimore, MD, USA

## Abstract

The demographic transition from high to low levels of mortality and fertility brings about changes that stretch the “arc of life,” making each stage of life longer and creating new ones—a phenomenon we call “the demographic stretch.” This stretch can transform societal structure, for example, by extending childhood, shifting working ages up, delaying marriage and childbearing, improving women’s status and equity, and pushing the burden of chronic disease and disability to older ages. Global health efforts must address the resultant economic and social changes.

The demographic transition, when countries shift from high to low levels of mortality and fertility, has been underway for 2 centuries, with differences in onset and pace across countries and regions of the world.^1^^-^^3^ Over the course of the transition, average life expectancy more than doubles, from about 40 years to 80 years, while the average fertility rate declines from about 7 children per woman to 2.5 children per woman—nearing replacement levels.

Living longer does not mean simply adding more years of decrepitude to the end of life. We posit that unprecedented increases in life expectancy stretch the “arc of life,” making each stage of life longer and creating new ones—a phenomenon we call “the demographic stretch.”

The clearest example of the demographic stretch is the transition from child to adult: the concept of “adolescence” was popularized only in the 20th century, and the term “teenager” was unknown before World War II. (G. Stanley Hall coined the term “teenager” in 1904 to describe the stage of life made possible by child labor laws and universal education, before youth faced the responsibilities of adulthood.^4^) Rather than work and marry, young people are more likely to go to college and postpone marriage, and sexual and reproductive activity may be likewise postponed or take on different guises—a phenomenon not yet fully captured by rigorous scientific studies.^5^ The total fertility rate (TFR) declines despite an increase in fecundity (due to earlier menarche and older age at menopause).^6^ Social and cultural changes tend to follow the demographic changes and need to be consciously articulated.

In this paper, we explore the social and cultural implications of this “demographic stretch” and the impact of different timing across generations and geographical regions using publicly available country data from United Nations agencies and other sources. We conclude the paper with recommendations for a research agenda to better understand the demographic stretch and its drivers and implications.

## THE DEMOGRAPHIC TRANSITION

For centuries, the world was relatively poor, with low life expectancy and high fertility rates. This changed with the demographic transition, which first began in France and then in the United States around the turn of the 19th century.^2^^,^^7^ In France, between 1750 and 2000 life expectancy more than tripled, from 25 years to 80 years.^8^ Similarly in the United States, life expectancy for whites nearly doubled over 150 years, increasing from 40 years in 1850 to 77 years in 2000.^7^ (Prior to the 20th century, data on life expectancy of blacks in the United States, which differed significantly from that of whites due to slavery, were scarce.) Declines in fertility accompanied increases in life expectancy. In France, the fertility decline began around the 1820s, with the TFR falling from about 4 children per woman in 1800 to 2.2 children per woman in 1930.^9^ The TFR in the United States fell from 7.0 in 1800 to 2.3 in 1940.^7^

The demographic transition occurred next in other parts of Europe, with dates of the initiation of fertility decline varying among countries. Fertility rates in Belgium, Germany, and Switzerland began to fall in the 1880s, while in Ireland and Spain fertility levels started to decline only around the 1920s.^9^ Life expectancy also increased. Today, more than half of babies born in rich nations will live to 100 years.^10^

**FIGURE 1:**
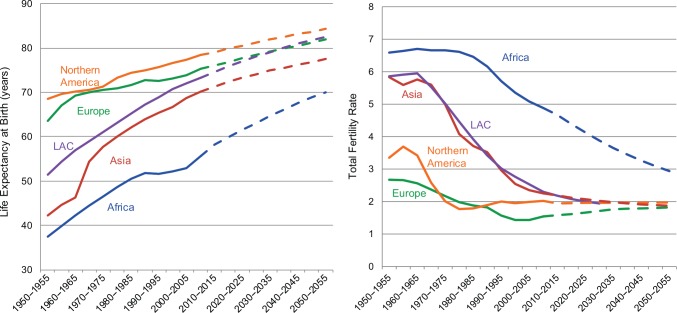
Life Expectancy at Birth and Total Fertility Rate, by World Region, 1950–2055 Abbreviation: LAC, Latin America and Caribbean. Dotted lines indicate projected data. Data Source: United Nations, 2012 (medium-variant fertility).^13^

Since 1950, regions at earlier stages in their demographic transitions have accelerated their progress with many approaching a “grand convergence”^11^ with richer nations: Africa, Latin America, and Asia have added 21, 24, and 29 years, respectively, to the life expectancy (Figure 1). Whereas in 1950 less than 1% of the world’s population lived in countries with life expectancy of 70 years or older, now over half do.^12^ There has also been a dramatic convergence in TFRs. The TFRs in Latin America and Asia have reached near replacement (2.2 children per woman). Africa’s TFR has declined since 1950, but it is still 4.7 today.^13^

The demographic transition in Africa, Asia, and Latin America is fast approaching that of richer nations.

However, life expectancy has not increased linearly and consistently in all parts of the world, and regional averages conceal a range of country differences. In the 1990s, life expectancy stagnated in Africa and actually decreased dramatically in some countries, mainly due to the HIV/AIDS epidemic. Eastern Europe also saw a stagnation or decrease in life expectancy in the 1990s.^12^ Likewise, there is significant variation in the TFR within regions and within countries. Despite this variation, people generally live longer and have fewer children, decoupling sexual activity from its reproductive function.

## STRETCHING THE ARC OF LIFE: SOCIAL AND CULTURAL IMPLICATIONS

Reductions in mortality across the span of life and changes in fertility patterns transform the population age structure, resulting in a net increase in median age.^12^ Early in the transition, progress is typically greatest in reducing under-5 mortality; reductions in older-age mortality follow.^12^ From 1950 to 2010, the median age increased from 30 years to 40 years in Northern America and Europe and from 20 years to 30 years in Latin America and Asia.^13^ Africa’s median age has stayed at about 18 or 19 years since 1950.^13^ By 2050, however, Africa is expected to reach a median population age of 25 years, and Asia and Latin America will converge with Northern America and Europe at about 40 years.^13^

The demographic transition results in a net increase in median age of populations.

As the median age rises and fertility declines, there are initially fewer dependent children relative to those in working productive ages. This creates an opportunity for increased economic growth known as the demographic dividend.^14^ This demographic dividend has been well documented in countries in East Asia, such as South Korea and Thailand,^15^ and it is now seen as playing a significant role in the ongoing economic success in developing regions of the world.^16^ This opportunity may diminish as median ages advance to 50 years or beyond unless workers increase their productivity or postpone retirement.^17^ The epidemiologic and economic implications of longevity are well studied.

Social and cultural structures also evolve in response to the demographic stretch and the disjunction and upheaval that might accompany it. We explore such social and cultural transformations through cross-sectional, ecological associations between longevity and various dependent variables, modeled using linear and logistic regressions while adjusting for gross domestic product (GDP) per capita.

### Childhood Is Lengthened

A clear, quick transition from childhood to adulthood is replaced with a more drawn-out progression through a series of sub-stages categorized by schooling (e.g., pre-Kindergarten through college) or by ages (e.g., preteen, teenager).^18^ In the stage of “emerging adulthood,” individuals have not yet achieved independence from parents due to increases in the number of years of schooling and delays in reaching other “adult” milestones such as marriage and entry into the formal workforce.^19^ Young adults’ continued dependence on their parents increases the cost of raising children.^20^ Recognizing these trends in the United States, the Affordable Care Act increased the age of health insurance eligibility in the family from 21 years to 26 years.^21^

### Education Is Prized More

**FIGURE 2:**
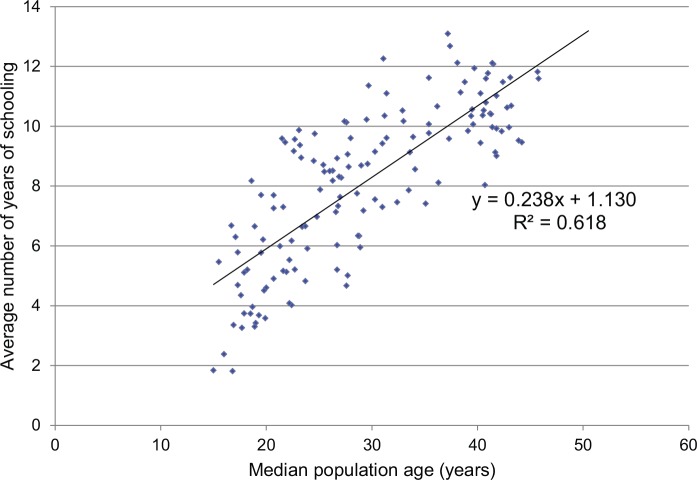
Relationship Between Average Years of Schooling and Median Population Age (138 Countries) Data Sources: Median population age in 2013 from The Kaiser Family Foundation Global Health Facts,^52^ and average years of total schooling (age 15+ in 2005–2011) are from the World Bank Open Data.^53^

Primary school becomes compulsory, more children complete high school, and more go on to college. In the United States, the percentage of the population completing college has grown from 5% in 1940 to 30% in 2009.^22^ An increase in a country’s median age by 1 year is associated with an average increase of 0.24 years of schooling (*P*<.001) (Figure 2). Even after controlling for log GDP per capita, an increase in a country’s median age by 1 year is associated with an average increase of 0.14 years of schooling (*P*<.001). Societies confront issues of equity in access to education, and public subsidies of education expand into later years of schooling. In more economically advanced countries, post-graduate education becomes more common and a prerequisite for a professional career.

### Working Ages Are Shifted Upward

Entry into the formal workforce and the start of a career are pushed back to older ages. This delay impacts family formation, which is often postponed to later ages as well. Increasingly, people form families and launch careers in their late 20s rather than their late teens, which was common at the start of the demographic stretch. With this transition, societies progressively protect childhood by establishing child labor laws and minimum working ages for full-time labor. These transformations occurred in North America and Europe in the late 1800s and early 1900s while efforts to end exploitative child labor practices continue in the developing world today.^23^

Entry into the workforce, along with family formation, is pushed back to later ages as a result of the demographic transition.

At the same time, as societies restrict child labor, the average age of retirement recedes to older ages. This can create disjunctions if retirement systems and health insurance benefits do not keep pace with longer life expectancies. For example, violent protests in Greece and elsewhere have broken out in recent years in response to proposals to increase the retirement age and cut pensions.^24^ In the United States, debate is ongoing regarding changing the national social security system that provides benefits to retired workers. Because of the increase in life expectancy, the system may need to raise the onset of retirement payments to later years in order to ensure its solvency.^25^

The upward shift in the working age calls for a redefinition of how we view “dependency.” Demographers classify children under age 15 as dependent, with the implication that once reaching age 15, children become independent. While this may have typified agrarian societies, it does not reflect the realities of today. Research using national transfer accounts data shows that for most all countries, individual levels of consumption outweigh production at ages well into the 20s, and in some cases into the early 30s.^17^ The threshold for old-age dependency, at times 60 years or 65 years, likewise, needs to be rethought as retirement is pushed to higher ages and as older people remain active and independent.

### Marriage Is Delayed While Non-Marital Childbearing and Cohabitation Increase

**FIGURE 3:**
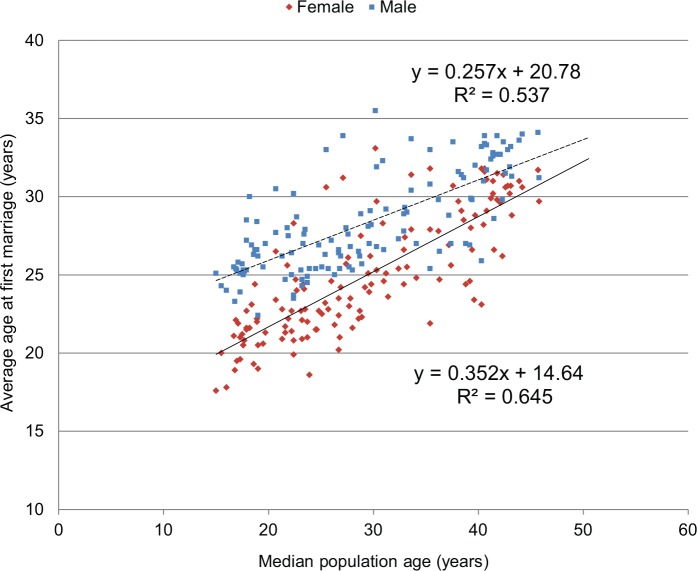
Relationship Between Median Age of the Population and Average Age at First Marriage (120 Countries) Data Sources: Median population age in 2013 from The Kaiser Family Foundation Global Health Facts,^52^ and average age at first marriage (in 2005–2011) are from the World Bank Open Data.^53^

Longer childhood, more schooling, decreased fertility expectations, and later entry into the workforce delay marriage for both men and women. In 1880s Russia, an unmarried woman of 24 years was considered a “spinster.”^26^ Today, a woman unmarried in her 20s would be commonplace in many parts of the world. In the United States, between 1950 and 2009, the median age at first marriage increased from 23 years to 28 years for men and from 20 years to 26 years for women.^27^ A similar trend has already been noted in many developing countries such as India,^28^ Côte d’Ivoire,^29^ and Senegal.^29^ As shown in Figure 3, there is a significant association between median population age and age at first marriage, which is deferred, on average, by 0.35 years for females (*P*<.001) and by 0.26 years for males (*P*<.001) as median population age rises by 1 year. When controlling for log GDP per capita, median age at first marriage is delayed by 0.16 years for females (*P*<.001) and 0.14 years for males (*P*<.001), on average, with each year increase in a country’s median age.

Cohabitation shifts from a taboo to a commonplace precursor or alternative to marriage. In the United States, the rate of cohabitation has increased from near zero 100 years ago to 33% in 1987 and to 50% in 2002.^30^ Global comparisons show that non-marital cohabitation is common in more developed regions, including the Americas, Europe, and Oceania, while it is still rare in other regions.^30^ With delays in marriage sometimes come increases in out-of-wedlock births. In Iceland, 66% of births are to unmarried women, and in Sweden this proportion is 55%.^31^ In the United States, about 40% of children are born to unmarried mothers; this proportion was just 5% in 1960.^31^^,^^32^ Having children within a cohabiting union becomes more common as cohabiting couples who become pregnant feel less pressure to wed. About 60% of out-of-wedlock births in the United States are to cohabiting couples.^32^

### Childbearing Is Delayed and Teenage Pregnancy Declines

**FIGURE 4:**
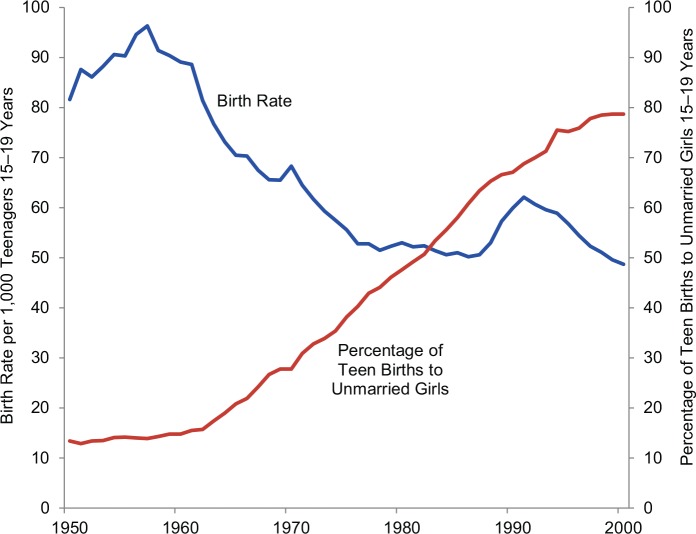
Births to Teenagers in the United States, 1950–2000 Source: Reproduced from the National Center for Health Statistics, 2001.^54^

The average age of a US mother at first birth increased from 21.4 years in 1970 to 25.0 years in 2006.^33^ Similar trends have occurred in other countries of the Organisation for Economic Co-operation and Development (OECD), where there has been a near-universal shift in age at first birth from the late teens to the late twenties.^33^ With greater access to contraceptives for youth, the rate of teen childbearing has declined significantly in Europe and North America (including among black and Hispanic teenagers); all the while, the proportion of teen births to unmarried youth has increased significantly—from less than 15% to 80% in the United States over the last 50 years (Figure 4).^34^ Sexual education and family planning have been critical to the decline in overall teenage pregnancy in the context of delayed marriage.

Age at first birth in the United States and other developed countries has increased to the late 20s.

In the United States in 1970, only 1 of 100 first births were to women 35 years and older; in 2006 this proportion was 1 of 12.^33^ Postponement of childbearing increases the mean length between generations and results in a changing “tempo” of childbearing.^35^ As postponements push childbearing into ages with lower fecundity, interest increases in induced ovulation methods, which produce greater chances for twins/multiple births. Rates of multiple births have increased substantially in many developed countries, including Canada, England and Wales, France, and the United States, over the past few decades in part because of increased maternal age and fertility treatment.^36^ These multiple births are associated with increased risk factors for infant mortality and long-term morbidity such as preterm birth and low birth weight. Increased maternal age is also associated with increased risk of congenital defects and gestational diabetes.^37^ However, the decrease in the number of children in families leads to higher investments in children. Parents with fewer children are able to spend more time with children and provide them with more resources and support.^38^

### Women’s Status and Equity Evolves

A central feature of these transitions is the changing role of women in society. With reductions in fertility, women experience greater opportunity for social participation. Gender gaps in education, work opportunities, income levels, and legal rights generally narrow. Control of fertility allows women to engage in educational, economic, and political activities and leads to changes in traditional gender roles.^16^^,^^38^^,^^39^ In the United States, women have outnumbered men in higher education since the late 1970s.^40^ Even Muslim-majority countries, which are often criticized for constraining women’s rights, have experienced concomitant increases in contraceptive use and equitable access to education over the past few decades.^41^ In Iran, where about 60% of women use a modern method of family planning and the TFR is 1.9, nearly equal proportions of males and females are enrolled in primary, secondary, and tertiary levels of school.^42^^,^^43^ In Egypt and Indonesia, the contraceptive prevalence rate and the male-to-female school enrollment ratio are about the same as in Iran, although fertility rates are slightly higher.^42^^,^^43^ Despite progress in gender equality in educational attainment, women in these countries lag behind men in workforce and political participation, highlighting that progress in all aspects of women’s empowerment is not automatic and immediate. In fact, we did not find a simple correlation between nations’ life expectancy (or their GDP) and the proportion of women working.

### Non-Traditional Lifestyles Are Increasingly Accepted

**FIGURE 5:**
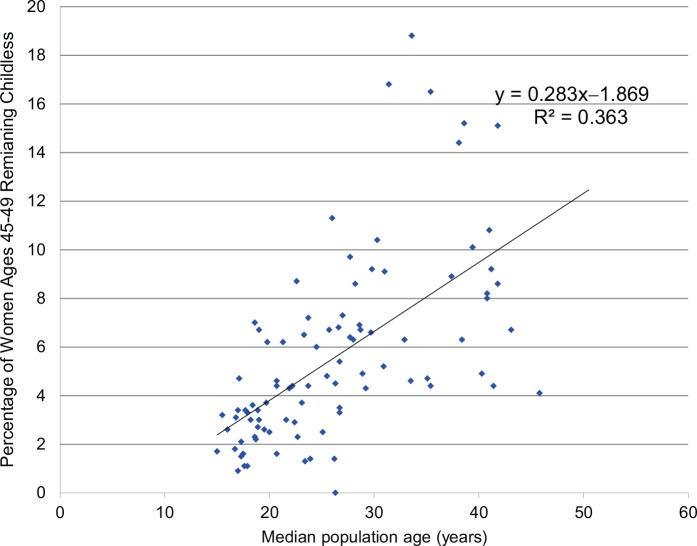
Relationship Between Median Age of the Population and the Percentage of Women Ages 45–49 Remaining Childless (92 Countries) Data Sources: Median population age in 2013 from The Kaiser Family Foundation Global Health Facts,^52^ and the percentage of childless women (in the most recent year available between 2000 and 2008) are from the United Nations, 2009.^55^

Not only are there overall delays in the initiation of life stages with the demographic stretch, there is also increasing freedom to deviate altogether from the traditional sequence of marriage followed by childbearing. Individuals and couples have increasingly chosen to remain childless. In some developed countries such as Australia, Germany, Italy, and the United States, the proportion of women in their late 40s who have no children has doubled over the past 30 years.^44^ As shown in Figure 5, countries with higher median population ages have higher rates of women ages 45–49 with no children (*P*<.001), although this relationship is not statistically significant after adjusting for log GDP per capita.

**FIGURE 6:**
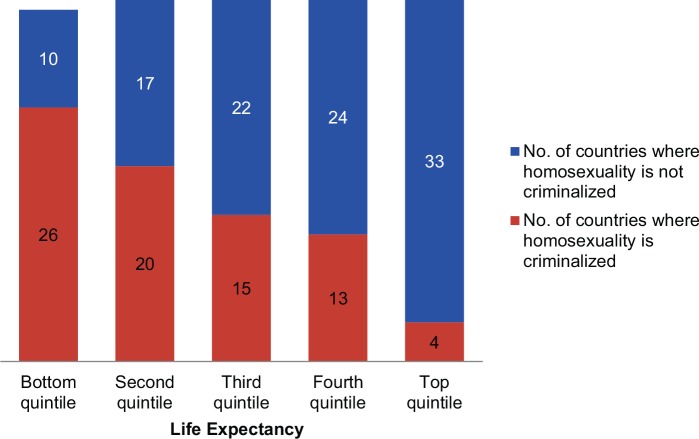
Relationship Between Life Expectancy and Criminalization of Homosexuality (182 Countries) Data Sources: Data on criminalization of homosexuality are from Clark, 2014,^45^ and data on life expectancy are from the United Nations, 2012.^13^

As sexual activity is decoupled from reproduction, people are more able to explore, discover, or assert different sexual orientations. Societies increasingly accept homosexuality and, more recently, have come to accept same-sex unions. Based on recent reports, 76 (41%) of 184 countries criminalize homosexuality.^45^ Figure 6 demonstrates that countries with the lowest life expectancies are least tolerant of homosexuality (72% of such countries criminalize homosexuality), and those with the highest life expectancies criminalize it the least (12% of such countries). In a logistic regression model, controlling for log GDP per capita, each 1-year increase in life expectancy is associated with a 12% decrease, on average, in the odds that a country criminalizes homosexuality (*P* = .006). The causal relationship here needs further exploration (e.g., what role do education, increasing secularization, or the decoupling of sexuality and reproduction have?). Clearly, tradition and political will can still strongly affect policy: 3 of the 4 high life expectancy countries that criminalize homosexuality have majority Muslim populations (Brunei, Lebanon, and Qatar). On the other hand, South Africa with a relatively low life expectancy has laws protective of sexual choice.

### Good Health Continues Through Middle Age and Often Beyond

The epidemiologic transition that follows the demographic one brings chronic diseases to the fore.^46^ The burden of chronic disease and disability increases^47^ but is pushed to older ages,^48^ and the functional status of individual octogenarians improves over previous generations. As new cohorts imprinted by the demographic stretch reach old age, sexual behaviors evolve since attitudes are more significant influences on sexual desire than biomedical factors.^49^ Many people remain sexually active after retirement: in the United States, 67% of 65- to 74-year-old men and 40% of women are sexually active.^50^ For 75- to 85-year-olds, 39% of men and 17% of women are sexually active.^50^

The burden of chronic disease and disability is pushed to older ages with the demographic transition.

## DISCUSSION

The world has undergone an unprecedented transition from high levels of fertility and mortality to much lower levels. The transition has not been uniform in onset or pace across countries, but it is nearly universal and progressing. It began 2 centuries ago in the countries of Europe and North America that are now considered “more developed” and has been followed in the past 50 years in many countries in Asia, Africa, and Latin America—and often at a much faster pace than in the more developed world. The transition continues today as child survival and fertility rates in poor countries converge with those of upper middle-income countries. This paper posits that these demographic shifts lead to a demographic stretch of the arc of life. Added years of life do not simply translate to added years of decrepitude. Rather, each stage of life is extended: we spend more years in childhood, more years as healthy adults, and more years in retirement. New sub-stages of life are added (e.g., “teenage”), and complex social changes emerge with some degree of predictability based on demographic transitions.

Added years of life do not simply translate to added years of decrepitude.

These shifts in the arc of life are not smooth or without economic and social upheavals: the staggered transition across generations, socioeconomic groups, and whole nations may create tensions due to the asynchronous evolution of norms in different groups or populations. At the macroeconomic level, they can require changes in the social contract, for example, in the need to raise the age at which retirement and health benefits become available. At the social level and in private life, we see impacts of the demographic stretch on child transitions to adulthood, transitions in family formation, and the role of women in society. The age of marriage moves from the teens to the late twenties, and sexual activity is decoupled from biological reproduction. The asynchronous nature of change means that moral and legal norms and the power relationships and attitudes they reflect may collide with the rapid demographic changes visiting the modern world, for example, in the recognition of gender equality or the acceptance of same-sex relationships.

The shifts in the arc of life may pose economic and social challenges that countries must address.

The preliminary associations presented here are just that: simple, descriptive, ecological observations that suggest the need for a research agenda to better understand the drivers of the demographic stretch of the arc of life changes, and of their implications. No single variable can explain complex sociocultural phenomena. The demographic stretch is the result of a complex interplay of increasing life expectancy, greater economic wealth, lower fertility rates, and the empowerment of women; changes in life expectancy and fertility correlate with such important variables as income and education, and sexuality measurement is far from perfect in much of the world. Rapid changes in information and communication technology and continued globalization will no doubt accelerate the changes. Panel data and time series analyses are needed to better understand the complex causal paths and interactions suggested by our hypothesis of a demographic stretch—in the context of a rich literature from psychology and anthropology. This growing understanding will help us develop a useful framework that will help policy makers and opinion leaders anticipate change and prepare for it.

The evidence presented here suggests the demographic stretch is a natural phenomenon followed rather predictably by changes in behaviors and social norms. It cannot by itself explain changing gender roles, sexual orientation, or the social acceptance of either phenomenon, but it does provide insight into the direction and drivers of change and is therefore a useful indicator of change. Countries that have undergone the demographic stretch cannot ignore their own historical evolution nor expect others to simply skip it as we do in more technical areas such as decreasing child mortality. In responding to countries where human rights appear to have been limited by local law or customs, being aware of the demographic stretch might help fast-track reform through more creative and effective approaches.

The demographic transition is well underway through much of the world. What might we expect to see as the arc of life continues to stretch? In countries where the transition is more advanced, we would expect to see more intergenerational conflict over, and changes in laws relating to, social welfare programs that benefit the elderly as declining numbers of younger people support increasing numbers of older people. We might see more countries entering a “birth dearth,” i.e., birth rates falling significantly below replacement levels, which Japan and other places are experiencing. While there will be economic consequences, these countries will still continue to see an increase in life expectancies and the demographic stretch. We look for further shifts toward later working ages as demand for labor increases relative to supply (and as life expectancies continue to increase), and possibly for the relaxing of and/or adjustments to immigration policies to address labor shortages.

In countries where the demographic transition is advanced, more intergenerational conflict may be expected.

At the other end of the wealth spectrum, low-income countries are witnessing the start of the transition. As their economies begin to expand, and with the faster spread of ideas through technology, we anticipate accelerated transitions over the next few decades—perhaps in a more compressed time period than observed elsewhere. Total fertility rates in the 48 least developed countries have been historically high but are beginning to fall, today at 4.3 compared with 5.7 children per woman just 40 years ago.^51^ Life expectancies have been historically low but are now rapidly increasing, today at 61 years compared with 44 years just 40 years ago.^51^ These countries have an aggregate population of 916 million, 40% of whom are less than 15 years old.^51^ For these countries, the social and economic changes that we have witnessed elsewhere and described in this paper could be amplified. We look for these countries to move at an accelerated pace toward social and political liberalization although with potential for a conservative backlash as social institutions and more traditional segments of a population resist these rapid changes.

With faster spread of ideas through technology, accelerated transitions might be expected in low-income countries at the start of the demographic transition.

## Notes

**Peer Reviewed**
